# Retromer in Synaptic Function and Pathology

**DOI:** 10.3389/fnsyn.2018.00037

**Published:** 2018-10-24

**Authors:** Lennart Brodin, Oleg Shupliakov

**Affiliations:** ^1^Department of Neuroscience, Karolinska Institutet (KI), Stockholm, Sweden; ^2^Institute of Translational Biomedicine, St. Petersburg University, St. Petersburg, Russia

**Keywords:** retromer, VPS35, synaptic vesicle, endosome, ionotropic receptor, G protein-coupled receptor, Alzheimer’s disease, Parkinson’s disease

## Abstract

The retromer complex mediates export of select transmembrane proteins from endosomes to the trans-Golgi network (TGN) or to the plasma membrane. Dysfunction of retromer has been linked with slowly progressing neurodegenerative disorders, including Alzheimer’s and Parkinson’s disease (AD and PD). As these disorders affect synapses it is of key importance to clarify the function of retromer-dependent protein trafficking pathways in pre- and postsynaptic compartments. Here we discuss recent insights into the roles of retromer in the trafficking of synaptic vesicle proteins, neurotransmitter receptors and other synaptic proteins. We also consider evidence that implies synapses as sites of early pathology in neurodegenerative disorders, pointing to a possible role of synaptic retromer dysfunction in the initiation of disease.

The retromer protein complex also referred to as retromer, is a critical component of the endosomal protein sorting machinery. This complex recognizes specific transmembrane proteins and exports them by forming tubules to promote transport. Of three endosomal export destinations (Figure 1A)—retrograde transport to the trans-Golgi network (TGN), recycling to the plasma membrane, and traffic to lysosomes—the former two are controlled by retromer (Seaman, 2012; McNally and Cullen, 2018). Retromer is composed of two main parts, the cargo-selection complex (CSC) and the tubulation module. The CSC consists of three largely globular proteins, VPS35, VPS26 and VPS29, named after the vacuolar protein sorting genes in yeast. The stability of the CSC depends on VPS35 and hence knockout/knockdown of this protein is commonly used to disrupt retromer function. The tubulation module comprises heterodimers of the BAR domain-containing sorting nexins SNX1/SNX2 and SNX5/SNX6 (Seaman, 2012; Mukadam and Seaman, 2015). Recent structural studies indicate that the CSC promotes tubule formation by directing the distribution of SNX proteins on the membrane surface (Kovtun et al., 2018).

**Figure 1:**
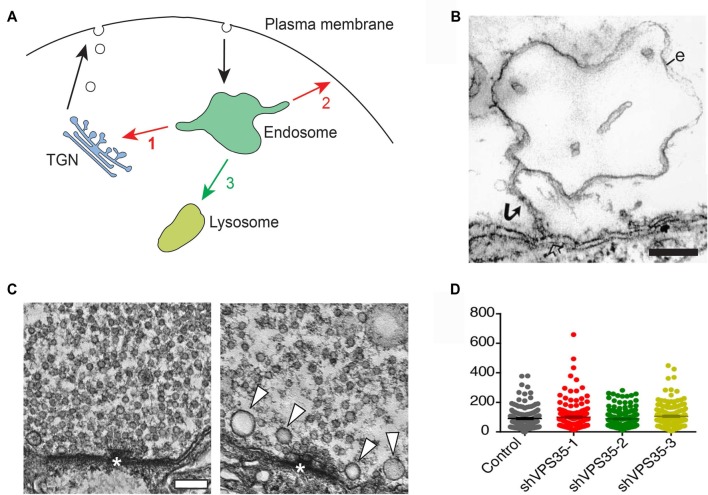
Retromer and the trafficking of synaptic vesicle proteins. **(A)** Endosomal trafficking pathways in animal cells. Proteins entering endosomes can be exported retrogradely to the trans-Golgi network (TGN; 1), or recycled back to the plasma membrane (2), or transferred to lysosomes via late endosomes for degradation (3). Retromer controls pathways 1 and 2. **(B)** ”False” endosome induced by synaptic activity at a presynaptic release site. The electron micrograph shows an endosome-like object (e) in a repetitively stimulated lamprey reticulospinal axon. Note the connection with the plasma membrane via a narrow stalk (arrow) revealing that the endosome-like object is in fact an invagination of the plasma membrane. Bar = 200 nm (reproduced from Gad et al., 1998 with permission). **(C)** Deletion of VPS35 in *Drosophila* alters synaptic vesicles in larval motor terminals. The left micrograph shows a control motor terminal. The right micrograph shows a terminal from a larva deficient in VPS35 and expressing a non-rescuing D620N mutant form of VPS35. Note the decreased number of synaptic vesicles and the occurrence of some large vesicles (arrowheads). Bar = 100 nm (reproduced from Inoshita et al., 2017 with permission). **(D)** Knockdown of retromer in mouse hippocampal neurons does not alter synaptic vesicles in nerve terminals. Quantitative comparison showing that the number of synaptic vesicles is similar in nerve terminals from control neurons and from neurons treated with either of three different shRNAs for VPS35 (reproduced from Vazquez-Sanchez et al., 2018 with permission: http://creativecommons.org/licenses/by/4.0/).

Retromer function depends on a set of accessory proteins. Among the most well characterized are SNX3 and Rab7a that mediate recruitment of the CSC to the endosomal membrane, and the GTPase activating protein TBC1D5, which acts on Rab7a to inhibit the recruitment process (Seaman, 2012; Seaman et al., 2018). The CSC also interacts with the WASH complex that mediates actin polymerization, which serves to constrain retromer cargo and/or signaling proteins at discrete endosomal regions. It may also take part in the scission of tubules (Seaman and Freeman, 2014). The WASH complex is associated with SNX1 via the DNAJ protein RME8 (Seaman and Freeman, 2014). Another accessory protein is the dynamin-like ATPase EHD. It is thought to stabilize endosomal tubules and take part in their scission (Zhang et al., 2012). In addition to those proteins, SNX27 has been implicated specifically in the trafficking of retromer cargos to the plasma membrane (Temkin et al., 2011). Contrary to yeast in which the CSC and the tubulation module are tightly associated, the two modules in animal cells are more loosely associated and can even act independently of each other, creating a greater functional diversity (Seaman, 2012, 2017).

## Retromer in the Adult and Developing Nervous System

Retromer components are ubiquitously expressed in the nervous system. The expression level varies between different brain regions and cell types (Wen et al., 2011; Wang et al., 2012; Lucin et al., 2013; Liu et al., 2014; Tsika et al., 2014; Appel et al., 2018). They occur in association with endosomes in the neuronal cell body and in processes (Wen et al., 2011; Bhalla et al., 2012; Mikhaylova et al., 2016). At synapses they are found in both the pre- and postsynaptic compartments (Jakobsson et al., 2011; Bhalla et al., 2012; Choy et al., 2014; Inoshita et al., 2017; Vazquez-Sanchez et al., 2018). Notably, in postsynaptic dendrites, retromer-bearing endosomes are associated with Golgi satellites, which mediate anterograde protein traffic from the ER as well as retrograde traffic from the plasma membrane (Mikhaylova et al., 2016).

In the developing brain the expression level of VPS35 peaks at stages P10–P15 and then declines to a low level that remains stable during adulthood (Wang et al., 2012). Accordingly, retromer plays a critical role in nervous system development. Full knockout of VPS35 causes death prior to neurogenesis, while heterozygous knockout, or *in utero* knockdown at a late embryonic stage, leads to impaired development of axons and dendrites (Wen et al., 2011; Wang et al., 2012; Tian et al., 2015). At least some of the developmental defects are due to loss of retromer present in microglia (Appel et al., 2018). The precise mechanisms by which retromer influence mammalian CNS development are not yet clear, but distinct retromer-dependent developmental signaling pathways have been identified in *Drosophila* and *C. elegans* (Wang and Bellen, 2015).

## Retromer and Presynaptic Protein Trafficking

Protein trafficking in presynaptic terminals is dominated by synaptic vesicle proteins, which are inserted into the plasma membrane upon exocytic neurotransmitter release and subsequently retrieved by endocytosis (Shupliakov and Brodin, 2010). Whether or not the retrieval is followed by an endosomal sorting step prior to vesicle re-use is a classical problem that has been discussed for decades (Heuser and Reese, 1973; Jähne et al., 2015; Milosevic, 2018). Endosome-like structures do indeed form in stimulated nerve terminals, but in many cases they represent plasma membrane invaginations (Figure 1B) or endosome-like objects resulting from ultrafast endocytosis rather than *bona fide* endosomes (Gad et al., 1998; Kononenko et al., 2014; Watanabe et al., 2014; Jähne et al., 2015; Gan and Watanabe, 2018). Compelling evidence for endosomal sorting of synaptic vesicle components is currently limited to the larval *Drosophila* neuromuscular junction. In this model, synaptic vesicle recycling has been shown to involve presynaptic endosomes, identified by their characteristic lipid and protein composition (Wucherpfennig et al., 2003; Uytterhoeven et al., 2011). Moreover, enhancement of the endosomal recycling route increases the sorting away of ubiquitin-tagged synaptic vesicle proteins from presynaptic terminals (Uytterhoeven et al., 2011).

Two studies of retromer function at presynaptic terminals have recently shed new light on the problem of endosome involvement in synaptic vesicle recycling. In the first study, Inoshita and co-authors examined the effect of deleting VPS35 in *Drosophila* (Inoshita et al., 2017). They examined the effect in mutant larvae that survived to a prepupal stage due to the supply of some maternal VPS35. Ultrastructural analysis of VPS35-deficient motor terminals revealed that the number of synaptic vesicles was reduced while their size was increased and was more variable (Figure 1C). The morphological changes correlated with an enhanced fatigue of synaptic transmission. These data thus corroborate the involvement of endosomes in synaptic vesicle recycling in *Drosophila* larvae, and provide the first evidence for a functional role of retromer in the synaptic vesicle cycle. In the second study, the effects of knocking down VPS35 in mouse hippocampal neurons was investigated (Vazquez-Sanchez et al., 2018). To circumvent developmental defects the knockdown was performed when synaptogenesis was essentially completed. Notably, ultrastructural analysis did not reveal any difference in the number of synaptic vesicles between control and VPS35-depleted nerve terminals (Figure 1D). Nor did the knockdown affect synaptic exo- or endocytosis, which were monitored with a pH-sensitive reporter (Vazquez-Sanchez et al., 2018).

In view of these apparently contradictory data it is unavoidable to speculate that the developmental stage is a critical factor. Studies in toad and mouse motorneurons, for example, suggest that synapse maturation can involve a switch from endosome-dependent to endosome-independent vesicle recycling modes (Zakharenko et al., 1999; Shetty et al., 2013). If this is true, the retromer system in mature nerve terminals can be assumed to serve other functions than to sort synaptic vesicle proteins. In this context it is interesting to note that Vazquez-Sanchez et al. (2018) detected VPS35 in some but not all hippocampal nerve terminals. Investigation of the role of retromer in different synapse types, and at different developmental stages will be of great interest for further studies.

Another presynaptic endosomal system of considerable physiological and pathological importance consists of signaling endosomes. Such endosomes take part in sorting and retrograde axonal transport of endogenous proteins like BDNF and its receptors TrkB and p75NTR, and exogenous agents like Tetanus toxin (Deinhardt et al., 2006; Shupliakov and Fernandez-Chacon, 2008; Surana et al., 2018). Notably, they are also enriched in proteins linked with neurodegenerative disorders (Debaisieux et al., 2016). Whether or not signaling endosomes utilize retromer currently remains an open question.

## Retromer and Neurotransmitter Transporters

The plasma membrane dopamine transporter (DAT) acts to terminate DA transmission primarily by mediating reuptake into dopaminergic presynaptic terminals. DA reuptake is affected by psychostimulants such as cocaine and amphetamine, and altered reuptake has been linked with different neuropsychiatric conditions (Sawa and Snyder, 2002; Kristensen et al., 2011; Sharma and Couture, 2014). The level of DAT at the presynaptic plasma membrane is finely tuned by endocytosis followed by either degradation or recycling back to the plasma membrane. Recently, Wu et al. (2017) showed that retromer plays a key role in DAT handling. Newly endocytosed DAT was observed to enter retromer-positive endosomes, and knockdown of VPS35 decreased DAT recycling leading to reduced plasma membrane levels. Moreover, the increase of plasma membrane DAT levels induced by cocaine (Little et al., 2002) could be linked with enhanced recycling of DAT out of retromer-positive endosomes (Wu et al., 2017). Whether other neurotransmitter transporters are sorted by retromer remains to be investigated.

## Retromer and Ionotropic Neurotransmitter Receptors

**Figure 2:**
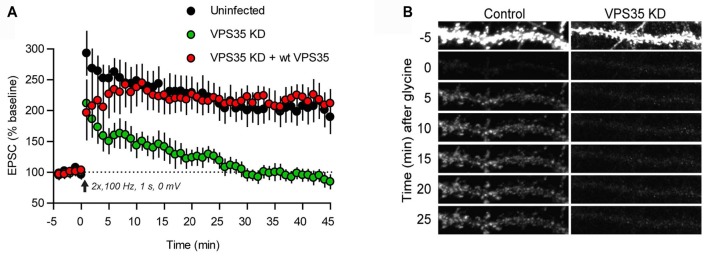
Retromer supports AMPA receptor trafficking during long-term potentiation (LTP). **(A)** Plot of the excitatory postsynaptic current (EPSC) amplitude during induction of LTP in control hippocampal slices (black dots) and hippocampal slices infected with lentivirus expressing an shRNA for VPS35 (green dots), and a further control, in which VPS35 expression had been restored (red dots). **(B)** Induction of chemical LTP causes incorporation of GluA1 receptors (tagged with a pH-sensitive reporter) in the dendritic plasma membrane of a hippocampal control neuron (left). The dendrite was photobleached prior to LTP induction to reveal the successive accumulation of GluA1 receptors. Knockdown of VPS35 (right) effectively inhibited receptor incorporation (reproduced from Temkin et al., 2017 with permission).

The postsynaptic compartment is a hotspot for trafficking of neurotransmitter receptors. With regard to ionotropic receptors detailed studies have primarily concerned glutamate receptors, which mediate most fast synaptic communication in the brain. In particular, the AMPA receptor subtype, made up of GluA1–4 subunits, has been thoroughly examined (Pick and Ziff, 2018). AMPA receptors are supplied to the postsynaptic membrane by two principal routes. One comprises constitutive delivery whereby receptors are inserted at extrasynaptic sites and then move into synapses by diffusion. The other is a triggered mechanism, which is used to insert receptors into the postsynaptic plasma membrane upon induction of long-term potentiation (LTP), and to remove them upon induction of long-term depression (LTD; Lüscher et al., 2000). In immature hippocampal neurons the constitutive delivery pathway depends on retromer. Thus, heterozygous knockout of VPS35 leads to a reduction of excitatory synaptic transmission along with a reduced amount of AMPA receptors in synaptosomes (Tian et al., 2015; see also Choy et al., 2014). Knockout of SNX6 or SNX27 similarly reduces AMPA receptor trafficking to the postsynapse (Hussain et al., 2014; Loo et al., 2014; Niu et al., 2017). In mature neurons, however, the role of retromer is restricted to the triggered pathway. Knockdown of VPS35 in the CA1 region of hippocampus at P21 does not affect basal synaptic transmission but causes an efficient blockade of LTP (Figure 2A; Temkin et al., 2017). Imaging of tagged AMPA receptor subunits suggested that retromer mediates the exit of AMPA receptors into “LTP-ready” vesicles that fuse with the dendritic plasma membrane (Figure 2B). The induction of LTD was not affected by retromer depletion (Temkin et al., 2017).

It should be noted that inhibitory synaptic transmission via GABA receptors is unaffected by retromer depletion even in immature neurons (Choy et al., 2014), thus suggesting a differential regulation of excitatory and inhibitory receptors.

## Retromer and G-Protein Coupled Neurotransmitter Receptors

G-protein coupled receptors (GPCRs) comprise a large and heterogenous group that induce a wide variety of intracellular signals, mainly via G-proteins or β-arrestin. Recent studies suggest that retromer plays a key role in the transduction of GPCR signals. This is partly due to the fact that GPCRs, unlike ionotropic receptors, act not only at the plasma membrane but continue to signal at intracellular sites, including endosomes and the TGN (Eichel and von Zastrow, 2018). The localization of GPCRs to these internal compartments typically gives rise to a slower and more prolonged response as compared to plasma membrane localization. Moreover, the type of signal can also be affected. For example, retromer-dependent localization of TSH receptors to the TGN activates a transcriptional response (CREB phosphorylation) that is not seen when the receptor remains at the plasma membrane (Godbole et al., 2017). Similarly, localization of β2 adrenergic receptors and dopamine D1 receptors to endosomes promotes transcriptional responses (Tsvetanova and von Zastrow, 2014; Varandas et al., 2016). In so far most studies of spatial factors and retromer in GPCR signaling have been conducted in cell lines, but future studies in neurons with many of their receptors located in distal dendrites and nerve terminals is likely to give a new level of insight into neuronal GPCR communication. The clinical importance of localized GPCR signaling has recently been underscored by the observation of a spatial signaling difference between opioid drugs and the corresponding native peptide ligands (Stoeber et al., 2018).

Retromer can also regulate the plasma membrane levels of GPCRs (including D1 and β2 receptors) by recycling them back from endosomes (Choy et al., 2014; Wang et al., 2016). Imaging studies in striatal medium spiny neurons have elegantly tracked the export of β2 receptors from retromer-bearing endosomes that move in the vicinity of postsynaptic densities (Choy et al., 2014). Moreover, GPCR signaling can be regulated by retromer in a more direct way. The CSC component VPS26, which is structurally similar to β-arrestin, can terminate GPCR signaling by displacing β-arrestin from the GPCR (Seaman, 2018).

## Links to Neurodegenerative Disorders

Retromer has gained wide interest in recent years due to its involvement in neurodegenerative disorders (Small and Petsko, 2015; Li et al., 2016; McMillan et al., 2017; Williams et al., 2017; Reitz, 2018; Vagnozzi and Praticò, 2018; Zhang et al., 2018). With regard to Alzheimer’s disease (AD), the first evidence came from protein profiling studies showing a reduction of VPS26 and VPS35 in brain regions affected by the disease (Small et al., 2005). Genetic studies have subsequently coupled AD with a number of retromer-associated proteins, including SNX1, SNX3, rab7a and SORL1/SORLA (Vardarajan et al., 2012; Lambert et al., 2013; Reitz, 2018). The latter is a retromer receptor that binds to and removes the amyloid precursor protein (APP) from endosomes (Eggert et al., 2018). Experimental studies have shown that depletion of VPS35 enhances amyloid β peptide (Aβ) production by prolonging the endosomal residence time of APP (Bhalla et al., 2012). Conversely, enhancement of retromer function with a pharmacological chaperone can reduce Aβ formation (Mecozzi et al., 2014), as can overexpression of SNX3 (Xu et al., 2018). The endosomal trafficking of the rate-limiting enzyme in Aβ production, BACE1, also depends on retromer (Wen et al., 2011; Wang et al., 2012; Toh et al., 2017).

Adding to the link between retromer and APP processing, indirect evidence also suggests an involvement of retromer in Tau pathology, which is another hallmark of AD. The delivery of cathepsin D to lysosomes is retromer-dependent and cathepsin D deficiency has been shown to aggravate Tau toxicity (Small and Petsko, 2015). Moreover, retromer stabilization can reduce pathology-associated Tau phosphorylation (Young et al., 2018).

**Figure 3:**
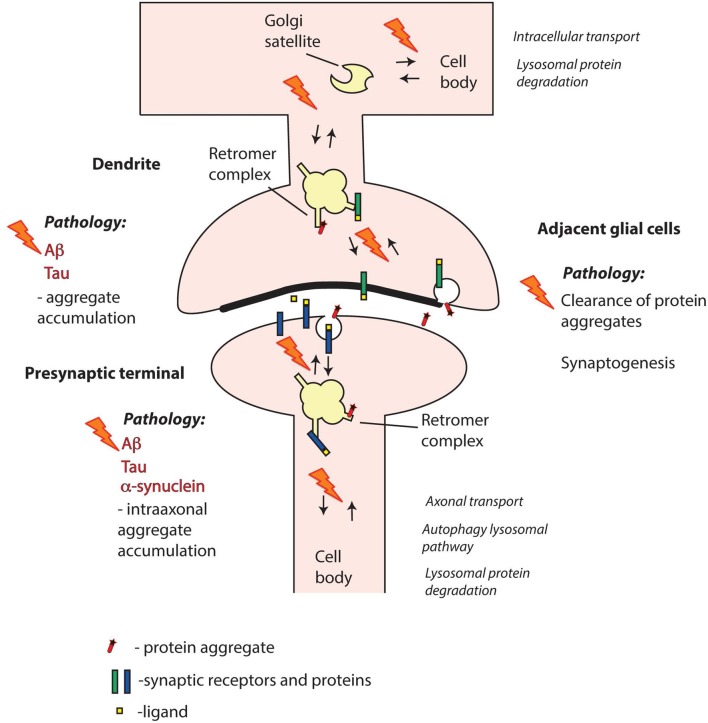
Retromer failure at different synaptic sites may contribute to neurodegenerative disorders. Aβ and Tau are key pathogenic proteins in Alzheimer’s disease (AD) that can impair both pre- and postsynaptic functions. α-synuclein is a presynaptic protein strongly linked with Parkinson’s disease (PD). Mutations or altered expression/processing of retromer components may result in impaired retromer function in pre- and/or postsynaptic compartments and perturbed protein degradation. Moreover, retromer dysfunction in adjacent glial cells, including both microglia and astrocytes, may contribute to decreased clearance of pathogenic proteins from the synaptic region. Retromer defects may also trigger aberrant phase separation of key proteins that can contribute to aggregate formation. Overall, impairment of retromer function at synapses may thus result in various defects in the handling of Aβ, Tau and α-synuclein leading to pathological aggregate formation.

In spite of the links with Aβ and Tau pathology, the precise role of retromer in AD pathogenesis is not fully clear. The question of whether synaptic retromer systems are involved remains open (Figure 3). Synapse loss is a hallmark of AD that occurs early in disease progression (Masliah et al., 2001; Scheff et al., 2006), and both Aβ and Tau pathologies have been linked with synapses. Thus, Aβ is produced locally at synapses (Dolev et al., 2013; Lundgren et al., 2014, 2015; Das et al., 2016; Schedin-Weiss et al., 2016), and Aβ oligomers exert toxic effects both pre- and postsynaptically (Ovsepian et al., 2018). Hyperphosphorylated and misfolded Tau oligomers accumulate at pre- and postsynaptic sites at early disease stages (Spires-Jones and Hyman, 2014; Tai et al., 2014), and different Tau forms can disrupt pre- and postsynaptic functions (Hoover et al., 2010; Ittner et al., 2010; Zhou et al., 2017; McInnes et al., 2018). The prion-like spread of Tau pathology between brain regions (Braak and Braak, 1991) appears to occur via synapses, and seed-competent Tau aggregates have been found enriched in synaptosomes (Chang et al., 2018; DeVos et al., 2018). Taken together, these observations point at synaptic retromer systems as possible players in early AD pathogenesis.

Genetic evidence implicates retromer also in Parkinson’s disease (PD). A missense mutation in VPS35, D620N, has been found to cause late-onset PD in several patient populations world-wide (Williams et al., 2017; Cui et al., 2018). The D620N mutation affects the interaction between VPS35 and the WASH complex, which has multiple effects on endosomal traffic (Seaman and Freeman, 2014; McMillan et al., 2017). The precise link with PD pathogenesis still remains unclear. It has, for instance, been suggested that retromer dysfunction may impair the clearance of α-synuclein aggregates, a hallmark of PD, either by impairing the delivery of cathepsin D (via the retromer receptors CI-MPR or SORLA) to lysosomes, or of autophagy-related protein 9a to autophagosome precursors (Follett et al., 2014; Zavodszky et al., 2014; Small and Petsko, 2015; Cui et al., 2018). It has also been suggested that connections between retromer and different PD-associated gene products, like LRRK2, Parkin and PLA2G6, are of importance, or that mitochondrial defects play a role (Small and Petsko, 2015; Williams et al., 2017; Lin et al., 2018; Williams et al., 2018).

Similar to the case with AD, some forms of PD are strongly linked with synapses, and primarily with the presynaptic compartment. Evidence from postmortem and neuroimaging studies in humans along with animal model data suggest that the degeneration of substantia nigra DA neurons may originate in their projections to striatum rather than in the cell bodies (Burke and O’Malley, 2013; Kordower et al., 2013; Laguna et al., 2015; Schirinzi et al., 2016; Pan et al., 2018; Soukup et al., 2018). Among the proteins that have been linked to early PD pathology, α-synuclein has been most extensively studied. This protein is normally accumulated in nerve terminals where it is associated with synaptic vesicles (Burré et al., 2017). Its different pathological forms—oligomers, protofibrils and aggregates—can exert toxic effects in nerve terminals (Burré et al., 2017; Bridi and Hirth, 2018). α-synuclein pathology may spread in the brain when it occurs in the extracellular space and it has been suggested that synapses are involved in this process via secretion and/or uptake of α-synuclein aggregates (Volpicelli-Daley and Brundin, 2018).

The composition and roles of the retromer system in nigrostriatal nerve terminals yet waits to be defined (apart from being implicated in DAT handling as discussed above). Its functional importance is supported by the observation that a mutation in VPS35, D620N, leads to altered DA turnover in striatum (Ishizu et al., 2016; Cataldi et al., 2018).

Other neurodegenerative diseases linked with retromer include Down syndrome, a variant of hereditary spastic paraplegia, and neuronal ceroid lipofuscinoses (Small and Petsko, 2015; Zhang et al., 2018). With regard to Down syndrome, the disease mechanism may be similar to that in AD as the expression of APP (located at chromosome 21) and Aβ production are enhanced. Moreover, the expression of miR 155 is enhanced causing a reduction of SNX27 expression that can both compromise synaptic glutamate receptor traffic (Wang et al., 2013) and interfere with APP processing (Zhang et al., 2018).

## Conclusions and Future Perspectives

Although the importance of retromer at synapses is beginning to become evident, the field is yet at an early stage and many questions remain to be answered. First and foremost, the scheme of endosomal cargo retrieval vs. degradation (Figure 1A) has been worked out in compact cell bodies and its correlates in distantly located synapses remain largely unexplored. Moreover, insights into neuronal retromer functions are in most cases limited to a handful of neuron types or to extrapolation from cell line studies. A clear priority is thus to expand the study to a broader set of neuron types. This is particularly true for presynaptic retromer systems of which the functions are least well understood. Another priority is to define more precisely the dynamic localization and composition of retromer systems and their accessory proteins in distinct types of synapses. High resolution imaging of these protein complexes at synapses under different conditions will be one of the challenges. Knowledge about putative synapse-specific accessory proteins will permit directed functional studies, and may also facilitate pharmacological development directed at e.g., GPCRs and neurotransmitter transporters.

As yet, direct evidence connecting pre- or postsynaptic retromer systems with pathology are not at hand but, as discussed above, there are many plausible links (Figure 3). Adding to these, another possible connection has recently emerged, which is related to phase separation of proteins (Gomes and Shorter, 2018). Recent evidences indicate that the functional organization of different proteins in the presynaptic (Milovanovic et al., 2018) as well as postsynaptic (Zeng et al., 2016) compartment depends on liquid-liquid phase separation. If phase separation would apply to intracellular pools of for example α-synuclein, or even Aβ and Tau, it may be envisioned that subtle defects in synaptic retromer-dependent protein clearance could initiate a seeding process eventually leading to transition from soluble proteins to insoluble aggregates.

Future experiments focused on the link between synaptic retromer systems and the synapse pathology in AD and PD may proceed along different lines. One may relate to improved knowledge about synapse-specific accessory proteins. It would, for example, be of key interest to identify negative regulators that could be used as drug targets. Another line may focus on retromer-stabilizing pharmacological chaperones (Mecozzi et al., 2014). Ways to target such agents to synapses could potentially enhance protective effects and reduce side-effects. Moreover, elucidating the molecular basis of the reduction of retromer components in sporadic AD (Small et al., 2005) may lead to new principles that address the basis of the synapse loss.

## Author Contributions

Both authors have contributed to ideas and writing of the manuscript.

## Conflict of Interest Statement

The authors declare that the research was conducted in the absence of any commercial or financial relationships that could be construed as a potential conflict of interest.
